# Infectious Disease: A Deadly MIF

**Published:** 2006-09

**Authors:** Tina Adler

Up to 2 million children die each year from malaria, with about half dying from malaria-induced anemia. Scientists aren’t sure why some malaria patients develop this life-threatening complication and others don’t. A study published in the 15 May 2006 *Journal of Experimental Medicine* suggests the blame rests in part on macrophage migration inhibitory factor (MIF), an immune cytokine produced by white blood cells.

The paper’s authors suspected that MIF might suppress bone marrow activity, because polymorphisms of the *MIF* gene increase susceptibility to different inflammatory and infectious disorders. “MIF appears to be part of an over-exuberant response on the part of the immune system in a number of diseases, so it was a logical choice for us to look at,” says coauthor Michael A. McDevitt, a hematologist at the Johns Hopkins University School of Medicine. The team infected mice that were genetically modified to lack the *Mif* gene with malaria parasites. About one-third of those mice survived, compared to only 9% of the normal mice.

MIF doesn’t act alone, the researchers discovered when they took progenitor cells from the bone marrow of mice and allowed the cells to grow both with and without MIF and two other immune factors, TNFα and IFNγ. Applied alone in low concentrations, none of the immune factors seriously damaged the bone marrow cells. “But when we added all three together at the same low levels, we witnessed a synergistic poisoning of bone marrow,” says McDevitt.

MIF probably prevents cells in the bone marrow from responding to erythropoietin, the hormone that triggers red blood cell production, says coauthor Richard Bucala, an immunologist and rheumatologist at Yale University School of Medicine. Some people’s immune system may make too much MIF in response to malarial infection. About 30% of the population in Africa, where malaria is rampant, produces excessive MIF protein, earlier studies have shown.

MIF isn’t the only cause of malarial anemia. The *Plasmodium* parasite destroys red blood cells, and the spleen also removes infected and even some uninfected cells. “The pathogenesis of [malarial] anemia has been a mystery for a long time,” says Peter J. Hotez, a parasitologist at George Wash*ington University. “This mouse* study adds to the evidence . . . that MIF impedes the production of red blood cells.”

Understanding the mechanisms behind malarial anemia could help direct future therapies. MIF is the key therapeutic target, because blocking it alone protects the cells, whereas blocking TNFα or IFNγ alone doesn’t, explains Bucala. Since the blood transfusions needed to treat severe anemia are often difficult for many poor families to find or afford, researchers hope to determine early on which children need immediate care to prevent the anemia.

To that end, Bucala and colleagues are collaborating on a project with Macha Mission Hospital in Zambia to assess the frequency of *MIF* polymorphisms in children with malarial anemia. They use a new, inexpensive method to identify the polymorphism in just 2 hours, letting them know whether a child will likely need a transfusion. In Zambia, children have a well-baby visit soon after birth, and it is possible every baby could get genotyped, says Bucala, who adds, “Then the mother would know that if her child develops fever in the rainy season, she should go to the hospital quickly.”

## Figures and Tables

**Figure:**
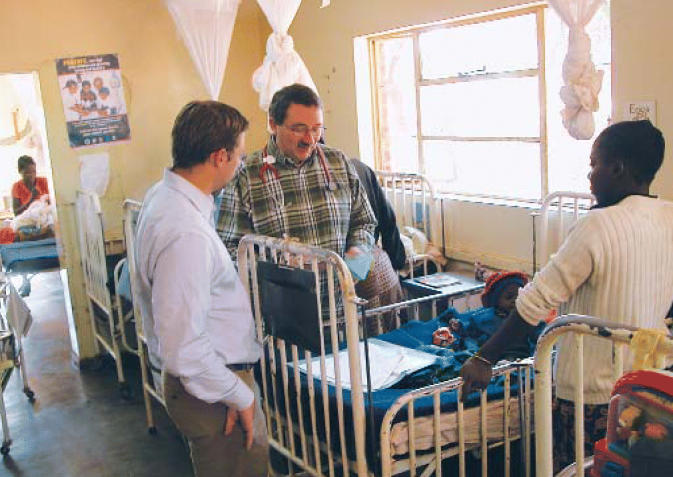
Unraveling a life-threatening complication A genotyping project at Macha Mission Hospital in Zambia, where director Phil Thuma (center) conducts pediatric rounds, aims to prevent malaria-induced anemia in children.

